# Primary Villoglandular Mucinous Adenocarcinoma of the Vulva

**DOI:** 10.1155/2017/1765460

**Published:** 2017-04-19

**Authors:** Akiko Matsuzaki, Masanao Saio, Noritake Kosuge, Hajime Aoyama, Tomoko Tamaki, Hirofumi Matsumoto, Naoki Yoshimi

**Affiliations:** ^1^Division of Pathology, Ryukyu University Hospital, University of the Ryukyus, Okinawa, Japan; ^2^Department of Tumor Pathology, Faculty of Medicine, University of the Ryukyus, Okinawa, Japan

## Abstract

Primary villoglandular mucinous adenocarcinoma of the vulva is rare tumor. We report a case of 68-year-old woman who developed this very uncommon malignant tumor. Immunohistochemical examination of this tumor revealed positive staining for Cytokeratin 20, Mucin 2, and CDX2, although Cytokeratin 7 and Mucin 6 were negative. This positive staining indicated the tumor enteric type characters. In order to exclude the possibility of the metastasis from another site, we thoroughly evaluated clinical data and extensively investigated the whole body. However, we could not detect any other tumors. The patient was treated by tumor resection. The patient remains free of disease 5 years after surgery.

## 1. Introduction

Primary adenocarcinomas of the vulva are rare and especially the villoglandular mucinous adenocarcinoma of intestinal type is rare variant of vulvar adenocarcinoma. In the 4th edition of WHO classification of tumors of female reproductive organs, primary villoglandular mucinous adenocarcinoma is defined as a primary invasive glandular epithelial tumor of intestinal type and the synonyms are cloacogenic carcinoma or cloacogenic adenocarcinoma [1]. In the published articles [2], intestinal type mucinous adenocarcinoma was also taken as a synonymous with villoglandular mucinous adenocarcinoma. Villoglandular mucinous adenocarcinoma arises in the surface epithelium of the vulva. Only few cases have been reported to date [1–12]. The possibility that those tumors may originate from cloacal remnants has been raised [2–12]. In fact, it has been mentioned that misplaced cloacal remnants could be found in vulva. There is the hypothesis that such misplaced remnants transformed into a tubulovillous adenocarcinoma of enteric type. But still the mechanisms of development of villoglandular mucinous adenocarcinoma in vulva are controversial. Here we report the case of this rare disease.

## 2. Clinical Presentation

A 68-year-old woman had a vulvar lump with mild local discomfort. She had no relevant medical history. She came to our hospital. On physical examination, a 4 cm nodular yellowish lesion on the vestibule under the orifice of the urethra was seen and vulvar biopsy was taken. The mass occupied the one-third of the vestibule from right side to left side. The pelvic examination showed no other mass. The chest roentgenograph, abdominal CT, and colonoscopy were within reference range. The specimen of the vulvar biopsy was diagnosed vulvar adenocarcinoma. Then the patient was treated by vulvar tumor resection. The tumor was surgically staged as a TisN0. She remains well and free of disease 5 years after surgery.

## 3. Pathological Findings

**Figure 1:**
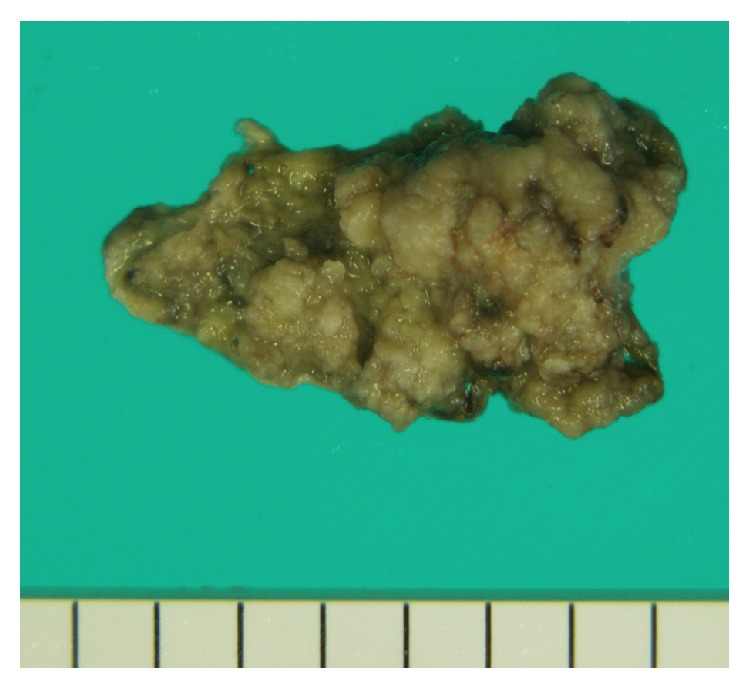
Surgical specimen showing the papillomatous, yellowish nodular lesion measuring 4 × 2 cm.

**Figure 2:**
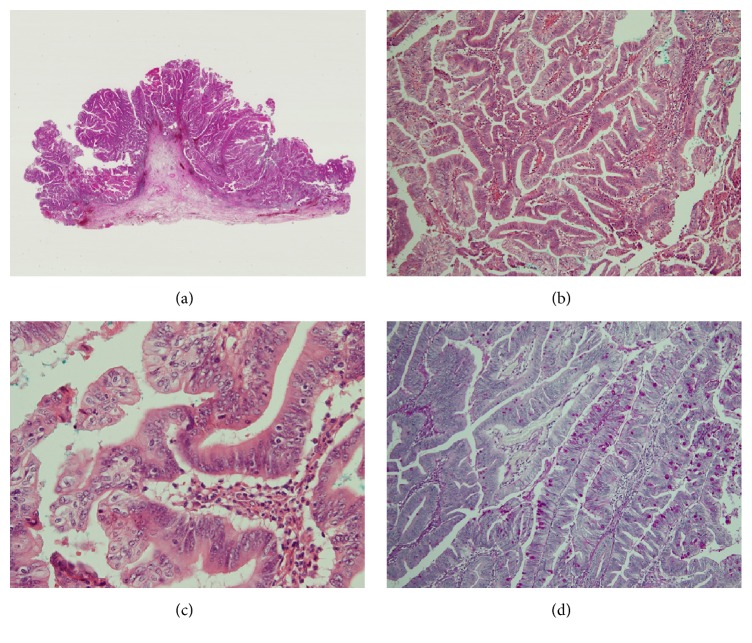
(a) Low-power view of the tumor shows papillomatous nodule. No tumor invasion was seen. HE staining. (b) Villous adenomatous tumor is observed. HE staining. (c) Tumor cells show high-grade nuclear atypia. (d) PAS staining shows positive staining for the goblet cells.

Grossly, the resected tumor from vestibule of vagina was papillomatous nodule, 4 × 2 cm in size and yellowish in color (Figure 1). Microscopic examination of paraffin-embedded sections (Figure 2) disclosed villous adenomatous tumor in continuity with the epidermis. The cells were columnar with ovoid nuclei, often stratified and sometimes proliferating into gland-in-gland pattern. The cells lost their normal basal polarity. The brush borders were observed. The small amount of goblet cells was seen but Paneth's cells were not detected. The tumor had a large proportion of mitotic cells and its resemblance to adenocarcinoma of the large intestine was striking. The high proportion of mitotic cells and resemblance to well-differentiated adenocarcinoma of the large intestine suggested that this neoplasm was adenocarcinoma rather than adenoma. No invasion or no microinvasion of the tumor cells was seen.

**Figure 3:**
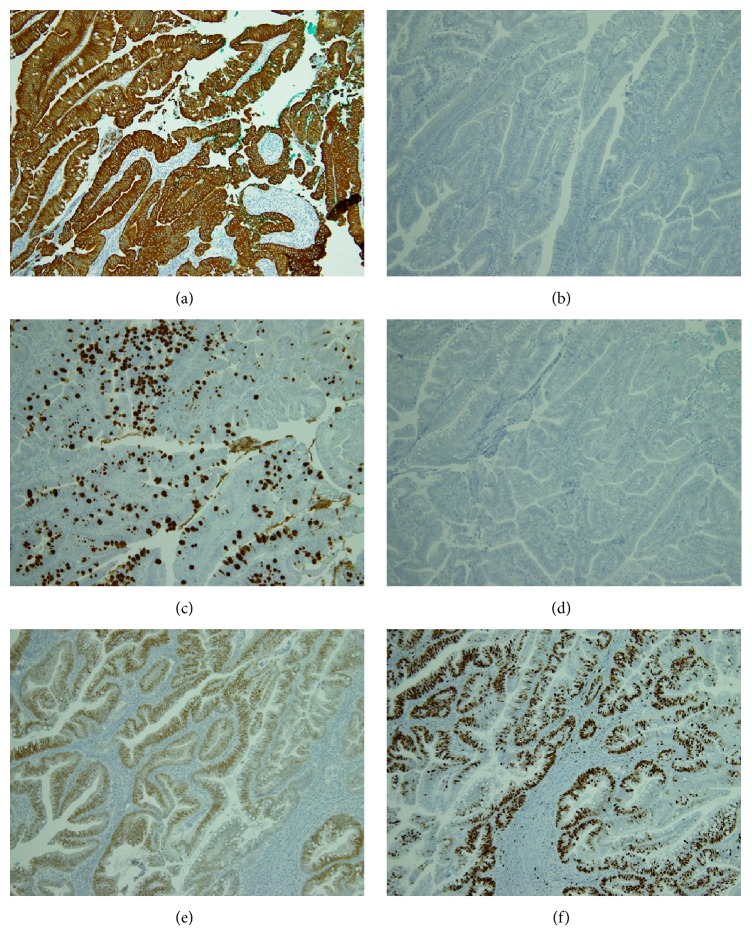
Immunohistochemical feature of the tumor cells. (a, b) Almost all tumor cells are positive for (a) CK20, but the cells are negative for (b) CK7. (c, d) Major proportion of tumor cells is positive for (c) MUC2, but expression of (d) MUC6 is negative. (e) CDX2 is positive for the nucleus of the tumor cells. (f) 40–50% of tumor cells are positive for MIB1.

Immunostains and PAS stains (Figures 2 and 3) were performed. Immunostains used a routine techniques with antibodies against the following antigens: Cytokeratin (CK) 7 (Dako, Glostrup, Denmark), CK20 (Dako), Mucin 2 (MUC2) (LAB VISION, Fremont, CA, USA), MUC6 (LAB VISION), CDX2 (BioGenex, San Ramon, CA, USA), and MIB-1 (Dako). In most neoplastic cells, prominent positive immunohistochemical staining for CK20, MUC2, and CDX2 was identified. CK7 and MUC6 did not demonstrate any reactivity. These data showed that these neoplastic cells had enteric type character. MIB-1 stain for nuclear antigen in proliferating cells showed a high proportion (more than 50%) of MIB-1 positive cells indicating an intense cellular proliferation. High proportion of MIB-1 positive cells suggested that this neoplasm was adenocarcinoma rather than adenoma. In [11], Karkouche et al. suggested that cellular atypia and high mitotic index indicate the intramucosal adenocarcinoma instead of adenoma. Pathologically our case was carcinoma in situ, and TNM stage was Tis.

## 4. Discussion

This is the case report of rare, vulva primary villoglandular mucinous adenocarcinoma in situ. This disease is more commonly known as adenocarcinoma of cloacogenic origin, enteric type adenocarcinoma, or cloacogenic adenocarcinoma [1–12]. Villoglandular adenocarcinomas are more frequent in the colon and rectum. The possibility that this lesion might be metastatic has been ruled out by a complete negative clinical workup. The precious mechanism of the development of enteric tumors in the female genital tracts is not known. However, since the lower vagina is derived from the urogenital tract, enteric neoplasia of the lower genital tract could develop from loci of gastrointestinal metaplasia from cloacal remnants. There are several reports of enteric type adenocarcinoma and adenoma in the vulva [1–13] (Table 1). These reports have supported the hypothesis that enteric neoplasia of the vulva could develop from loci of cloacal remnants. Similar enteric type adenoma and adenocarcinoma of the vagina and cervix were published [14–24]. Also this hypothesis is supported by several reports of villous adenoma and enteric type adenocarcinoma of the urologic system such as the bladder and urethra [25, 26]. Other possible mechanisms of enteric type tumor developed in the female genital tract are (1) intestinal metaplasia occurrence in tissue of Mullerian origin or (2) congenital ectopic intestinal epithelium of the urogenital tract as a result of embryological error. The use of immunohistochemical techniques has opened new perspectives in studying the primary villoglandular adenocarcinoma of the vulva. In general, the staining pattern of CK7−/CK20+ is the greatest proportion in colorectal carcinoma. And it is our case. Also, mucin pattern, MUC2+/MUC6−, suggested the colorectal carcinoma mucin pattern [27]. CDX1 and CDX2 are aberrantly expressed in intestinal metaplasia. The expression of CDX1 and CDX2 is closely associated with the expression of MUC2. In our case, both MUC2 and CDX2 were strong positive. These data clearly show that the character of this villoglandular mucinous adenocarcinoma is colorectal type. The histologic differential diagnosis of this case should include other types of vulva adenocarcinomas, such as adenocarcinoma of mammary gland type, adenocarcinoma of sweat gland type, and Bartholin gland carcinoma. Adenocarcinoma of mammary gland type has been reported as mammary-like ductal carcinoma or lobular carcinoma, morphologically and immunohistochemically different from our case. Sweat gland type adenocarcinoma and Bartholin gland carcinoma are also morphologically different from our case. These adenocarcinomas are deeply infiltrative and in situ carcinoma should be in these glands.

In all described villoglandular mucinous adenocarcinomas of vulva [1–12], the clinical behavior of this rare malignant neoplasm seems to be rather indolent, and patients are generally doing well, after either radical vulvectomy or wide local excision. Until we have further experience, tumor location, size, and microscopic appearance should determine the type of surgery. Good cooperation between clinician and pathologist is required for the determination of type of surgery. Bilateral inguinal lymph node dissection has been performed in the reported cases [5, 10] and no lymph node metastasis was reported. Despite apparent low risk of metastasis, we should discuss whether ipsilateral or bilateral inguinal lymph node dissection is required or not, because our knowledge for this tumor is still limited due to experience.

In conclusion, both pathologists and clinician should be aware of the existence of this rare tumor. However, more cases are needed to fully understand its origin and to establish its long-term prognosis.

## Figures and Tables

**Table 1:** Reported cases of enteric type tumor in female lower genital tract.

Authors	Age	Location	Histology	Immunohistochemistry	Follow-up	Ref.
Tiltman and Knutzen	50	Vulva	Adenocarcinoma	Not done	1 year	[3]
Kennedy and Majmudar	54	Vulva	Adenocarcinoma	CEA− CK+	10 years	[4]
	63	Vulva	Adenocarcinoma	Not done	4 years	
Ghamande et al.	80	Vulva	Adenocarcinoma	CEA+	19 months	[5]
	67	Vulva	Adenocarcinoma	CEA+	17 months	
Willén et al.	57	Vulva	Adenocarcinoma	CEA+, CK+	26 months	[6]
Rodriguez et al.	69	Vulva	Adenocarcinoma	CEA+, CK7+, CK20+, ER−PR−	36 months	[7]
Zaidi and Conner	43	Vulva	Adenocarcinoma	CEA+, CK+	18 months	[8]
Liu et al.	49	Vulva	Adenocarcinoma	Not done	24 months	[9]
Dubé et al.	58	Vulva	Adenocarcinoma	CK7+, CK20+, CEA+	16 months	[10]
Cormio et al.	58 42	Vulva Vulva	Adenocarcinoma Adenocarcinoma	CK7+ CK20+/−	Death 39 months	[2]
Karkouche et al.	31	Vulva	Adenomas and adenocarcinoma	Adenomas; CK7−/CK20+ Adenocarcinoma; Not done		[11]
Vitrey et al.	57	Vulva	Adenocarcinoma	CK7−CK20+ CDX2+, CA125+/−	17 months	[12]
Musella et al.	66	Vulva	Tubulovillous adenoma	Not done	N/A	[13]
Ciano et al.	72	Vagina	Villous adenoma	Not done	6 months	[14]
Ulbright et al.	5	Vagina	Papilloma	Not done	12 months	[15]
Fox et al.	35	Vagina	Enteric adenocarcinoma	Not done	N/A	[16]
	59	Vagina	Enteric adenocarcinoma	Not done	N/A	
Mortensen and Nielsen	43	Vagina	Tubulovillous adenoma	CEA	N/A	[17]
Nagar et al.	45	Vagina	Adenocarcinoma	CK+, CEA+, p53+, ER−, PR−,	18 months	[18]
Mudhar et al.	56	Vagina	Adenocarcinoma	CK7−, CK20+, CEA+	18 months	[19]
Werner et al.	67 45	Vagina Vagina	Adenocarcinoma Adenocarcinoma	CK7−, CK20−, CEA+ CK7+, CK20−, CEA−	14 months 23 months	[20]
Dubé et al.	64	Vagina	Adenocarcinoma	CK7+, CK20+	18 months	[21]
Lee et al.	61	Vagina	Tubulovillous adenoma	CK7+, CK20+, p53±	N/A	[22]
Tjalma and Colpaert	55	Vagina	Adenocarcinoma	CK7+, CK20+,	N/A	[23]
van Wessel et al.	68	Vagina	Adenocarcinoma	CEA+, CK20+, CK7+	25 months	[24]

Ref.; reference. N/A; not available.
